# Hypoxia-induced vasculogenic mimicry formation in human colorectal cancer cells: Involvement of HIF-1a, Claudin-4, and E-cadherin and Vimentin

**DOI:** 10.1038/srep37534

**Published:** 2016-11-21

**Authors:** Wen Li, ShaoQi Zong, Qi Shi, HongJia Li, Jian Xu, Fenggang Hou

**Affiliations:** 1 Oncology Department of Shanghai Municipal Hospital of Traditional Chinese Medicine affiliated to Shanghai TCM University, Shanghai, 200071, China; 2 Mental diseases Department of Shanghai Municipal Hospital of Traditional Chinese Medicine affiliated to Shanghai TCM University, Shanghai, 200071, China

## Abstract

Vasculogenic mimicry (VM) plays an important role in colorectal cancer (CRC) metastasis, and both hypoxia and the epithelial-mesenchymal transition (EMT) are necessary for VM. In this study, HIF-1α expression was upregulated in the VM-positive CRC cell line HCT-116 and thereby affected the expression of the EMT-related markers Claudin-4, E-cadherin (E-cd) and Vimentin(VIM). SB431542 and U0126EtOH, which can inhibit of EMT were used to treat HCT-116 and HCT-8 in these experiments. Both of the inhibitors had significant effect on EMT markers and the formations of VM in CRC cells. In addition, knockdown of HIF-1α in the HCT-116 cells inhibited their capacity for VM. Our study reveals a regulatory role for HIF-1α in VM and suggests that targeting either HIF-1α or EMT may be a valuable strategy for the elimination of CRC metastasis.

Colorectal cancer (CRC) is the third most common cancer worldwide, and most patients are diagnosed with advanced disease^1^,^2^. Indeed, the accepted tenet underlying tumor survival has been that a blood supply is required to sustain growth and to metastasize^3^. It was believed that only endothelial cells could form blood vessels. However, a blood vessel’s rigid cellular identity has been challenged with the observation that tumor cells also form extravascular networks in melanoma. Several lines of evidence have indicated that malignant melanoma cells not only produce various angiogenic factors to promote the development of tumor vasculature from pre-existing blood vessels but also directly form tumor vasculature via vasculogenic mimicry (VM), a process by which tumor cells line up to form functional blood vessels independent of vascular endothelial cells^4^. We and others have proposed that tumors, including CRC, may develop a vascular network through angiogenesis, as well as through alternative pathways such as VM and the formation of mosaic vessels. Thus, vasculogenic genes and cells may be rational targets for anti-CRC therapy.

Epithelial cells can acquire a mesenchymal phenotype through a process known as epithelial-mesenchymal transition (EMT), which is a cellular plasticity process whereby epithelial cells down-regulate epithelial characteristics, such as cell-cell contacts and expression of E-cadherin, while simultaneously acquiring mesenchymal properties including a fibroblast-like morphology, increasing motility, and up-regulating mesenchymal markers such as vimentin^5^. Twist1, which binds to the E-box consensus sequence motifs in the promoters of its target genes, induces EMT by repressing E-cadherin and up-regulating the expression of mesenchymal markers^6^. Given that endothelial cells possess mesenchymal properties, the process of EMT is similar to that of VM. However, it is still unclear whether EMT is associated with VM or if EMT directly causes VM.

It has been reported that the expression of hypoxia-inducible factor-1α (HIF-1α) is associated with VM in primary gallbladder cancer, melanoma and hepatocellular carcinoma^7^,^8^,^9^. Twist1, the upstream regulatory molecule of EMT, is also induced by hypoxia during the process of VM^10^. Recently, it has been shown that hypoxia promotes vasculogenic mimicry by inducing EMT in ovarian carcinoma^11^. However, it is still unclear whether EMT is associated with VM or if EMT directly causes VM in CRC.

For these reasons, we used *in vivo* and *in vitro* assays to explore the mechanisms by which the HIF-1α/EMT pathway regulates VM in CRC. The data may offer a theoretical basis for the development of targeted CRC therapies.

## Materials & Methods

### Experimental animals

We confirmed that the Ethical Committee of the Shanghai University of Traditional Chinese Medicine approved all the experiments described in this paper. All experiments were performed in accordance with the official recommendations of the Chinese Community Guidelines. A total of 48 BABL/c nude male mice (18 ± 2 g) raised under specific pathogen-free conditions were purchased from Shanghai Slac Laboratory Animal Co. Ltd (Shanghai, China; animal license number SCXK (Shanghai) 2008–0016); they were fed in the experimental animal room of Putuo Hospital affiliated to Shanghai University of Traditional Chinese Medicine (Shanghai, China, animal laboratory license number, SCHK (Shanghai) 2007–0005). Sixteen animals were randomly allocated into 2 groups prior to the experiment. Human HCT-116 and HT-29 colorectal cancer cells were used to establish xenografts. Cells were re-suspended at a density of 1 × 10^7^ cells/ml, and the resulting suspensions (0.1 ml/10 g body weight) were injected subcutaneously. After 12 days, tumor nodules were palpable. On day 21, the mice were killed by cervical dislocation, and the tumors were removed, fixed in 4% paraformaldehyde (pH 7.2) and embedded in paraffin. Tumor sections were then stained with CD31 in order to observe tumor angiogenesis, and the number of vessels was enumerated in each of ten high-power fields. In addition, PAS staining was also performed to visualize channels formed by VM. The density of PAS-positive vessel networks was estimated using a photomicroscope (Leica, Germany), and for each tumor, 5 microscopic fields per slide in each of 5 slides were analyzed. Finally, slides were also stained for laminin, a component of the basement membrane of VM channels.

### Cell lines and culture

The human CRC cell lines HCT-116 and HT-29 (both originally isolated from a primary colonic tumor) were purchased from the Chinese Academy of Science. HCT-116, LS174T and HCT8 cells were cultured in RPMI-1640 (Gibco, USA) medium with 10% fetal bovine serum (FBS) (Gibco, USA), whereas HT-29 cells were cultured in McCoy’s 5A(Gibco, USA) medium with 10% FBS. All cells were cultured with 100 μg/mL of streptomycin (Invitrogen, Carlsbad, CA, USA) in a humidified incubator (Thermo Fisher Scientific Inc., Waltham, MA, USA)^12^ at 37 °C with 5% CO_2_. The EMT inhibitor U0126-EtOH(SELLECK, S1102, USA) and SB431542(SELLECK, S1067, USA).

### CD31/PAS double staining

For CD31/PAS double staining, CD31(Cell Signaling, USA, dilution 1:2000) immunohistochemical staining was performed first. Specifically, after application of the diaminobenzidine chromagen, slides were rinsed with distilled water, treated with 0.5% periodic acid solution for 10 min, and rinsed again with distilled water for 2–3 min. Next, the slides were incubated with Schiff solution for 15–30 mins in the dark, rinsed with distilled water, and finally counterstained with hematoxylin.

### Three-dimensional cultures

The formation of vascular channels was assessed using *in vitro* 3-D cultures composed of rat tail collagen type I. First, the 6-well plates were pretreated with a certain percentage of collagen for 30 minutes. Then, 4 × 10^5^ tumor cells were plated onto the surface of the collagen and incubated at 37 °C. Additionally, for in-gel methods, the tumor cells were mixed with Matrigel, which was then allowed to polymerize. Filtered CoCl_2_ was added to the medium in the hypoxic group 48 h after polymerization.

### HIF-1α Gene Silencing

HCT-116 cells were transfected with a validated short hairpin RNA (shRNA) targeting the HIF-1α sequence, 5′-GCAAUAGACAAGGACAUAATT-3′ (Shanghai GenePharmaCo., Ltd China). Transfections were performed using Lipofectamine™ 2000 reagent (Invitrogen, USA) according to the manufacturer’s protocol, and the cells were incubated at 37 °C for 48 hours before analysis. Then, the expression of HIF-1α mRNA was detected by quantitative PCR, and protein expression was analyzed by western blot.

### Real-time quantitative PCR

Total cellular RNA was isolated using TRIzol reagent (Invitrogen Life Technologies) according to the manufacturer’s protocol. RT reactions were performed using the Takara kit according to the manufacturer’s instructions. Quantitative PCR was performed using the SYBR green system (Takara). He forward primer for HIF-1α was 5′-CATCTCCATCTCCTACCCACAT-3′, and the reverse primer was 5′-ACTCCTTTTCCTGCTCTGTTTG-3′. The forward primer for human vimentin was 5′-GAAG

AGAACTTTGCCGTTGAAG-3′, and the reverse primer was 5′-ACGAAGGTGACGAGCCATT-3′.

The forward primer for human E-cadherin was 5′-GTCTCTCTCACCACCTCCACAG-3′, and the reverse primer was CAGACAGAGTGGGGAAAATGTA-3′. The forward primer for Claudin-4 was 5′-AAGTGACAGGGTGTGGTGGT-3′, and the reverse primer was 5′-CAGACAGAGTGGGGAAAATGTA-3′. The forward primer for TGF-β1 was 5′-CTGGCGATACCTCAGCAAC-3′, and the reverse primer was 5′-TAAGGCGAAAGCCCTCAAT-3′. The forward primer for Fn1 was 5′-GACCGAAATCACAGCCAGTAG-3′, and the reverse primer was 5′-CATCTCCCTCCTCACTCAGC-3′. Data are expressed as gene expression level relative to β-Actin (forward primer, 5′-AGCGAGCATCCCCCAAAGTT-3′; reverse primer, 5′-GGGCACGAAGGCTCATCATT-3′). All experiments were performed in triplicate, and a no-template reaction was used as a negative control. Relative mRNA expression levels were calculated using the 2^−ΔΔCt^ method.

### Western blot analysis

Cells were lysed in 10 volumes (w/v) of lysis buffer. After centrifugation, the supernatant was collected, and the protein concentrations of each sample were quantified. Equal amounts of total protein were separated by 12% SDS-PAGE and then transferred to a PVDF membrane. The membrane was then incubated overnight at 4 °C with primary rabbit anti-human monoclonal antibodies to HIF-1α (Abcam, UK; dilution 1:500), E-cd (Cell Signaling, USA, dilution 1:1000), Claudin-4 (Santa Cruz, USA, dilution 1:1000), and vimentin (Afinity, USA; dilution 1:1000), as well as rabbit anti-human polyclonal antibodies to β-actin (Bioworld, USA; 1:500). After incubation with an HRP-conjugated secondary antibody at room temperature for 2 h, protein bands were visualized using enhanced chemiluminescence (ECL) and detected using the Bio-Imaging System.

### Immunohistochemistry (IHC)

Tumor tissues were fixed in 4% paraformaldehyde for 48 hours, embedded in paraffin and sectioned into 4 μm slides. Tissue sections were then processed by de-paraffinization, rehydration through an alcohol gradient, peroxidase clearing, antigen retrieval and blocking, antibody binding, DAB staining, washing with distilled water, hematoxylin staining, niacin alcohol differentiation, dilute ammonia bluing, incremental graded alcohol dehydration, rinsing in xylenes, and mounting with a conventional resin. Slides were then incubated overnight at 4 °C with the following primary antibodies: rabbit-anti-human HIF-1α monoclonal antibody (Abcam, Cambridge, MA, USA; dilution 1:50), rabbit-anti-human E-cadherin monoclonal antibody (Cell Signaling, USA, dilution 1:100), Claudin-4 (Santa Cruz, USA, dilution 1:100), and vimentin (Afinity, USA; dilution 1:100). Secondary biotin-conjugated antibodies were used at 1:200. Positive tan staining for CD31 and E-cadherin was visualized with a light yellow or tan substrate and cell nuclei were counterstained with hematoxylin. Five random fields were selected per section and analyzed under 400X magnification. Staining was assessed with the following scale: no staining: 0 points; light brown: 1 point; brownish yellow: 2 points; and dark brown: 3 points. In addition, percentages of positively stained cells were also scaled: positive cells ≤5%: 0 points; 6–25%: 1 point; 26–50%: 2 points; and ≥75%: 3 points. Points for staining and percentage were multiplied to yield a 10-point scale: 0 points: negative (−), 1–3 points: weakly positive (+); 4–6 points: positive (++); and 7–9 points: strongly positive (+++)^13^.

### *In vitro* migration and invasion assays

HCT-116 and HT-29 cell invasion was assayed using 24-well Transwells with 8-μm-pore polycarbonate membrane inserts (Corning, NY, USA) and Matrigel (BD Biosciences) according to the manufacturer’s instructions. Briefly, 20 μl of Matrigel (1:5 dilution) was added to each insert. Then, 100 μl of a cell suspension containing 3 × 10^5^ cells was transferred to the upper chamber and allowed to incubate for 24 h. The filters were then stained with hematoxylin, and cells that appeared on the lower surface of the filter were counted in five random high-magnification microscope fields. Each experiment was performed in triplicate.

### Wound healing assay

Cell motility was assessed using a scratch assay. The width of the wound was measured after 12 and 24 hours, and the speed of wound closure was determined at each time point by calculating the wound width relative to that at 0 hours. Each experiment was performed in triplicate.

### Statistical analysis

Results were expressed as the means ± SD, and SPSS 18.0 was used for all statistical analyses. qRT-PCR data were analyzed using the paired Student’s t-test, whereas statistical analysis of other results was determined using one-way ANOVA. P < 0.05 was considered statistically significant.

## Results

### *In vitro* VM capacity of colorectal cancer cells is associated with invasiveness, migration and motility

**Figure 1:**
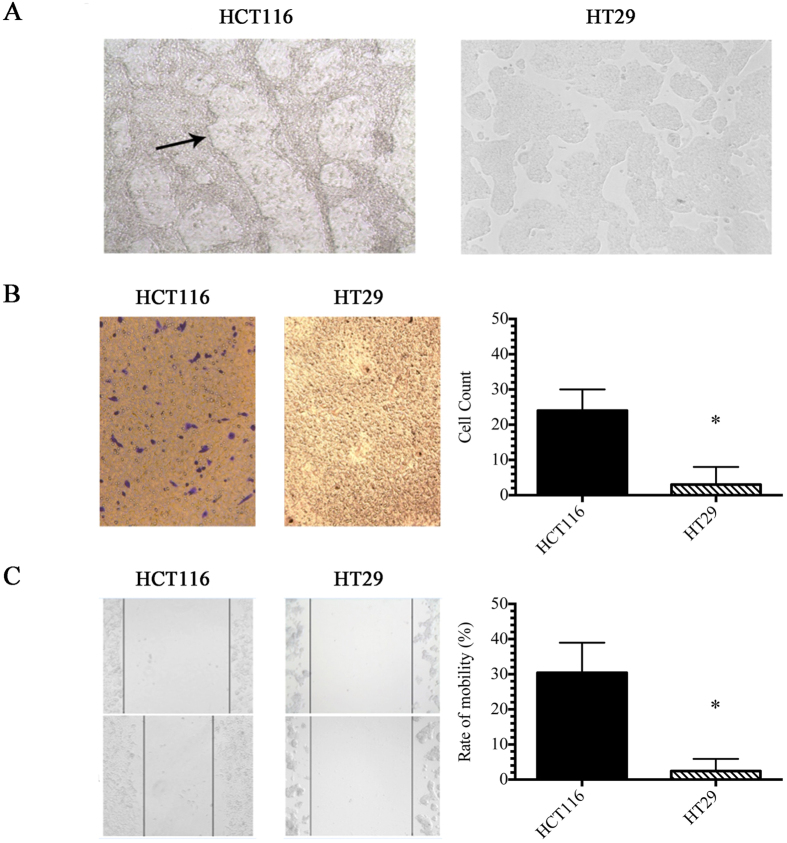
*In vitro* VM capacity of colorectal cancer cells is associated with invasiveness, migration and motility. (**A**) The formation of vascular channels in different CRC cell lines. HCT-116 cells could form typical pipe-like vascular networks when cultured on a three-dimensional matrix, whereas HT29 cells could not. Black arrows point to networks formed via VM. All scale bars indicate 100 μm. (**B**) For the invasion assay, HCT-116 cells were significantly more invasive compared to the control group (*P < 0.05). (**C**) In the wound healing assay, a significant difference in the migration speeds of the HCT-116 and HT29 cells was observed. The arrows signify the different motilities of each cell line (n = 3) (*P < 0.05).

The formation of vascular channels indicative of VM was tested using a 3-D culture system composed of rat tail collagen type I^4^. Two human CRC cell lines with different metastatic potentials were used, including a highly-metastatic (HCT-116) and a poorly-metastatic cell line (HT-29)^14^. HCT-116 cell VM was first observed at 24 h after seeding, and channel formation was completed by 6 days. However, HT-29 cells did not form vascular channels by VM (Fig. 1A). Next, cell invasion was assayed using Transwell chambers and serum-free media without the addition of a chemoattractant. After 24 h, a significant number of HCT-116, but not HT-29, cells had invaded the Matrigel, (Fig. 1B). Furthermore, as shown in Fig. 1C, quantitative analysis of a wound healing assay revealed significant differences between the wound healing speeds of HCT-116 and HT-29 cells.

Taken together, these results indicate that VM-positive HCT-116 cells were highly invasive, exhibiting both high cell motility and migration, while VM-negative HT-29 cells were less invasive. These results show a correlation between *in vitro* VM capacity and metastatic potential in CRC cells.

### Analysis of HIF-1α and EMT expression in VM-positive and VM-negative cell lines

**Figure 2:**
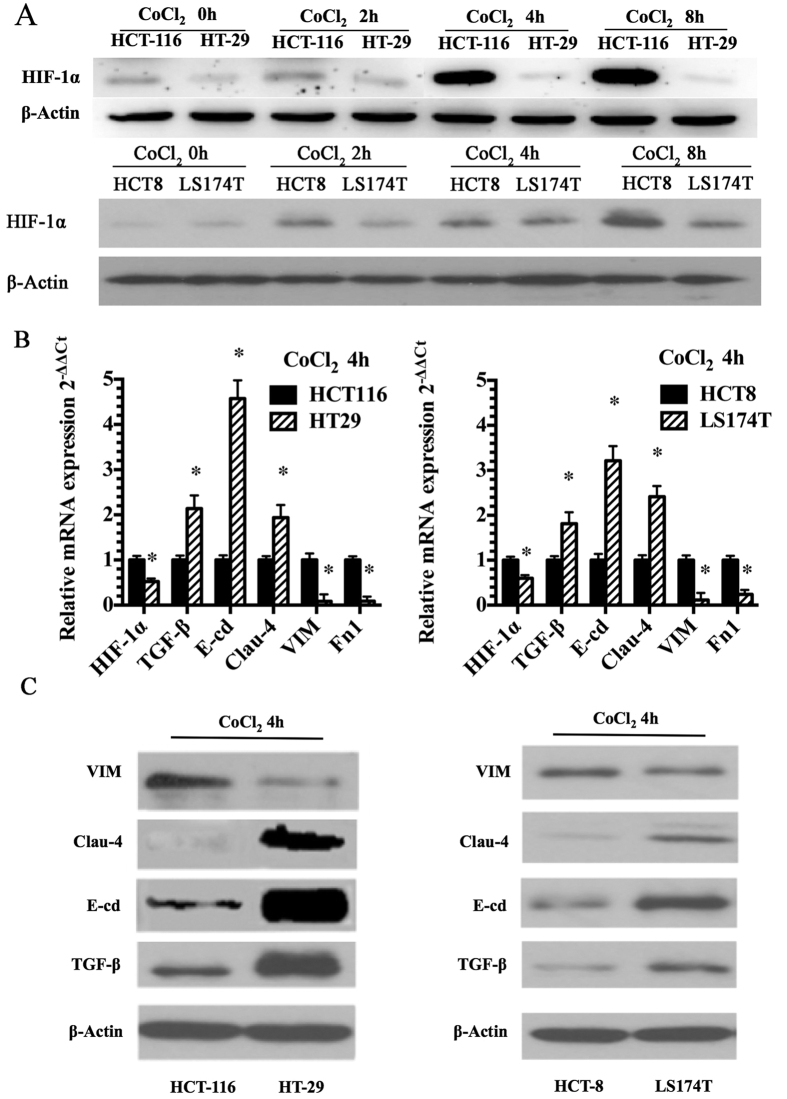
Analysis of HIF-1α and EMT expression in VM-positive and VM-negative cell lines. (**A**) CRC cells were incubated for 0–8 h under either normoxic or hypoxic (CoCl_2_) conditions, and the levels of HIF-1α protein were analyzed. (**B**) mRNA expression levels of HIF-1α, Fn1, vimentin (Vim), TGF-β1, Claudin-4 and E-cd by qRT-PCR in CRC cells. (**C**) Protein expression levels of HIF-1α, Fn1, vimentin (Vim), TGF-β1,Claudin-4 and E-cd by WB in CRC cells. *P < 0.05. The results are presented as fold change ± SE of at least three independent experiments.

The hypoxic microenvironment of tumors affects the metabolism, angiogenesis, and survival of cells orchestrated by HIF-1α, depending on tissue specificity. Hypoxic conditions were simulated *in vitro* using cobalt chloride (CoCl_2_), and cells were incubated for 0–8 h in either normoxic or hypoxic conditions. To determine the expression levels of HIF-1α, we analyzed the levels of HIF-1α protein in two CRC cell lines using western blotting. Both cell lines showed a significant increase in HIF-1α protein expression under hypoxic conditions (Fig. 2A). We then detected the mRNA expression levels of HIF-1α and several EMT markers in a panel of CRC cell lines using qRT-PCR. As shown in Fig. 2B, HCT-116 and HCT8 cells exhibited high expression levels of HIF-1α, Fn1 and vimentin (Vim), whereas HT-29 and LS174T cells exhibited low expression levels of these markers. However, the reverse was true for the markers TGF-β1, Claudin-4 and E-cd. Specifically, we found that HCT-116 and HCT8 cells exhibited low expression levels of these three markers, whereas HT-29 and LS174T, exhibited high levels. Moreover, Vim expression levels in HCT-116 and HCT8 cells were higher than those in HT-29 and LS174T cells. The trends for TGF-β1, Claudin-4, Vim and E-cd protein expression in HCT-116, HCT8, HT-29 and LS174T cells agreed with their respective levels of mRNA expression (Fig. 2C).

### EMT regulated in VM formation

**Figure 3:**
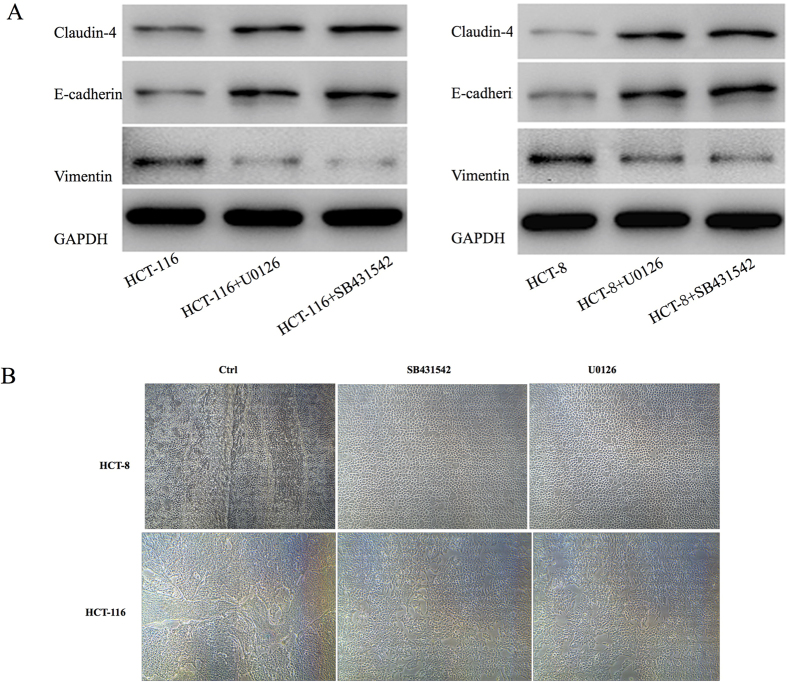
EMT regulated in VM formation. SB431542(10 μM) and U0126EtOH (8 μM), which can inhibit of EMT were used to treat HCT-116 and HCT-8 in these experiments. (**A**) Protein expression levels of EMT markers E-cd, Claudin-4 and Vim in CRC cells. *P < 0.05. (**B**) The VM formation in CRC cells after treated with EMT inhibitor.

In order to demonstrate the EMT regulated VM formation in CRC, SB431542 and U0126EtOH, which can inhibit of EMT were used to treat HCT-116 and HCT-8 in these experiments. Protein expression levels of EMT markers E-cd, Claudin-4 and Vim in CRC cells were detected by western blot. As shown in Fig. 3A, both of the inhibitor had significant effect on EMT markers. What’s more, the formations of VM in CRC cells were distinctly weakened after treated with EMT inhibitor (Fig. 3B). That is to say, EMT regulated VM formation in CRC. HIF-1α induced changes in EMT will be illustrated in the follow experiments.

### HIF-1α induced changes in EMT gene expression levels and altered VM in HCT-116 cells

**Figure 4:**
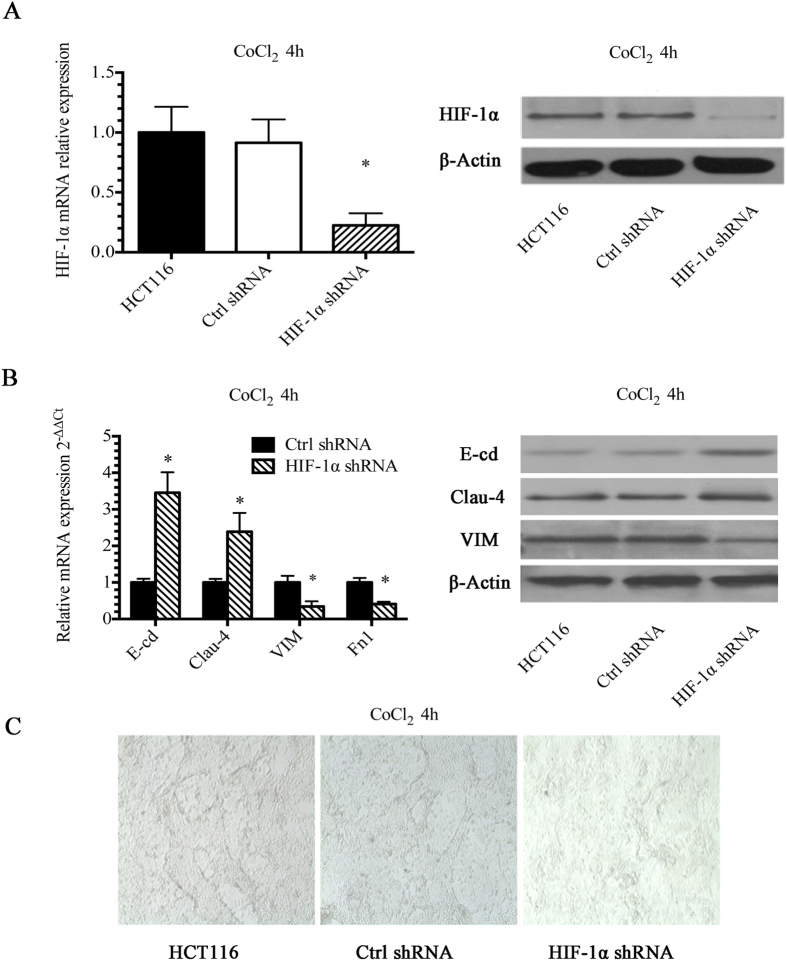
HIF-1α induced changes in EMT gene expression levels and altered vascular network formation in HCT-116 cells. (**A**) shRNA-HIF-1α was used to knock down the expression of HIF-1α. HIF-1α expression significantly decreased. (**B**) mRNA expression levels of the EMT markers E-cd, Claudin-4, Vim and Fn1 were significantly downregulated following HIF-1α knockdown. (**C**) HIF-1α shRNA significantly decreased VM under hypoxic conditions. *P < 0.05.

To further investigate the role of HIF-1α in the formation of vascular channels under hypoxic conditions, an shRNA construct (herein referred to as shRNA-HIF-1α) was used to knock down HIF-1α in HCT-116 cells. As shown in Fig. 4A, this construct significantly reduced HIF-1α expression. We also observed significant changes in the mRNA and protein levels of the EMT markers E-cd, Claudin-4, Vim and Fn1 following HIF-1α knock down (Fig. 4B). Furthermore, as expected, shRNA-HIF-1α significantly reduced the VM capacity of HCT-116 cells under hypoxic conditions relative to control cells (Fig. 4C). Taken together, these results indicate that hypoxia-induced VM is dependent upon HIF-1α expression.

### VM is associated with HIF-1α and EMT markers in CRC xenograft tumors

**Figure 5:**
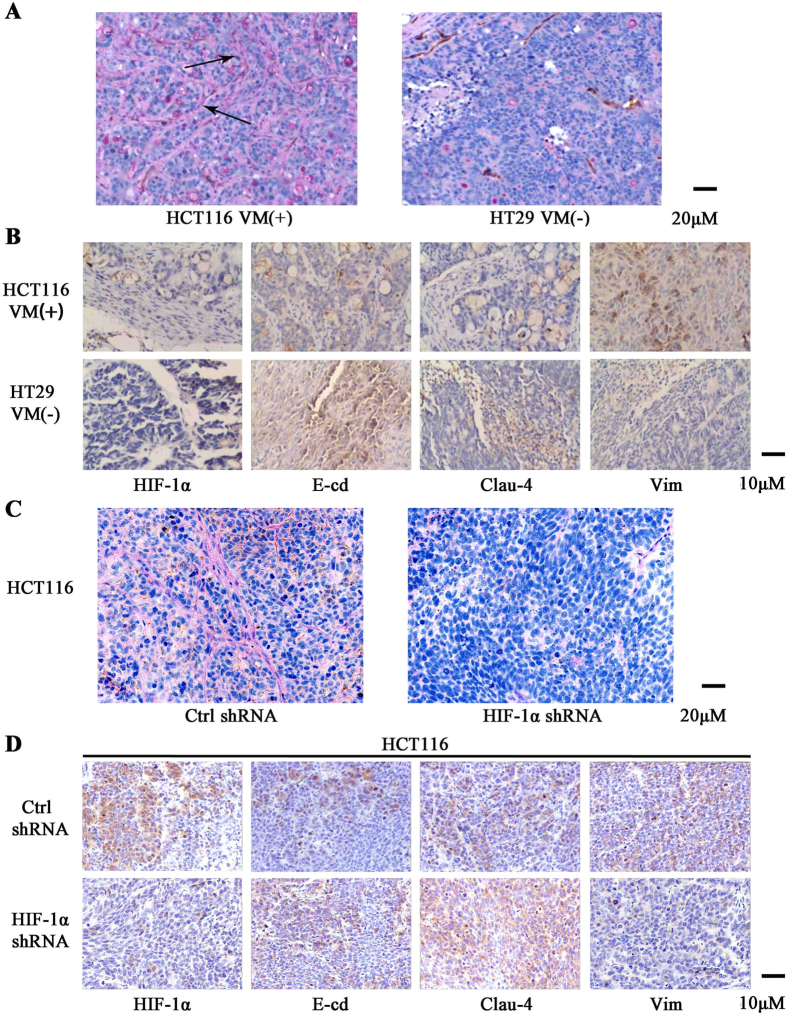
VM is associated with HIF-1α and EMT markers in CRC xenograft tumors. (**A**) IHC analysis of CRC xenografts showed that HCT-116 cells could form vascular networks by VM formation, whereas HT-29 cells did not. (**B**) The correlation between HIF-1α and E-cadherin, Claudin-4 and vimentin expression in CRC xenograft tumors. (**C**) IHC of CRC xenografts showed that HCT-116 could form vascular networks by VM, whereas the HCT-116 HIF-1a knock down cell line could not. (**D**) The correlation between HIF-1α and E-cadherin, Claudin-4 and Vimentin in the HCT-116 HIF-1a knock down cell line in tumor xenografts.

*In vivo* data showed that HCT-116, but not HT-29 cells, formed vascular channels (Fig. 5A). In addition, the formation of these channels was associated with increased expression of HIF-1α and vimentin, as well as reduced expression of E-cadherin and Claudin-4 in CRC xenograft tumors. Moreover, knock down of HIF-1α in HCT-116 tumor xenografts prevented VM and reduced gene expression changes implicated in the process of EMT (Fig. 5C and D).

## Discussion

Vasculogenic mimicry (VM) is the phenomenon where cancer cells mimic endothelial cells by forming blood vessels^15^. Tumor blood vessel formation is caused not only by angiogenesis dependent on host endothelial cells but also by VM, which plays an equally substantial role in this process^16^,^17^,^18^. VM, which refers to a process whereby tumor cells directly line up to form blood vessels, was first reported in melanoma by Hendrix and colleagues in 1999^4^. It was described as the unique ability of highly aggressive tumor cells to differentiate into multiple cellular phenotypes, obtain endothelial-like characteristics, and form vessel-like structures to obtain blood supply either actively or passively. Hence, it is suggested that the presence of tumor cell-lined channels may provide a potential new route by which metastases may leave the tumor to reach distant sites, using the exchange flow between the VM channels and normal vascular system. These findings explain why a class of drugs once heralded as a game-changer in cancer treatment was less effective than hoped^19^,^20^. This study was not the first in which researchers suggested that cancer cells could make their own blood vessels.

The induction of EMT in human mammary epithelial cells results in the acquisition of mesenchymal traits and the expression of stem cell markers^21^. Previous data show that cancer cell lines underwent morphological EMT-like changes (more fibroblastoid morphology and loss of cellular cohesiveness) under hypoxic conditions. Hypoxia led to an increase in migration, invasion and is critical for cell plasticity and VM formation^22^. Hypoxia (or induction of HIF-1α) mediates the behavior of important factors within the tumor microenvironment that induce EMT and promote a stem-like phenotype^23^. EMT is the main process that promotes VM in cancer, and VM has been associated with metastasis in CRC and reduced survival^24^. In this study, we provide evidence that VM is associated with HIF-1α expression, alterations in the levels of EMT markers, and metastasis in CRC. In addition, the use of shRNA-HIF-1α showed that the down-regulation of HIF-1α reduced VM in HCT-116 cells and the expression of the EMT markers E-cadherin, Claudin-4, Vim and Fn1. Concomitantly, our *in vivo* data support that VM capacity is significantly impacted by HIF-1α expression and EMT. Taken together, our data imply that hypoxia may directly cause VM by inducing EMT.

To our knowledge, this is the first report discussing the relationship between EMT, HIF-1α and VM using VM-positive CRC cells as a model. HIF-1α plays an important role in the development of VM, but further studies are needed to support this result. Most of the research conducted regarding EMT focuses on the invasion and metastasis of cancer cells. Taken together, our results indicate that the combined use of angiogenesis- and VM-targeting therapies may be an effective cancer treatment. Identification of individual EMT molecules that regulate VM in CRC is the focus of our ongoing work.

## Additional Information

**How to cite this article**: Li, W. *et al*. Hypoxia-induced vasculogenic mimicry formation in human colorectal cancer cells: Involvement of HIF-1a, Claudin-4, and E-cadherin and Vimentin. *Sci. Rep.* **6**, 37534; doi: 10.1038/srep37534 (2016).

**Publisher’s note:** Springer Nature remains neutral with regard to jurisdictional claims in published maps and institutional affiliations.
